# Fortified Human Milk Compared with Unfortified Human Milk in Preterm Low Birth Weight Neonates: A Systematic Review and Meta-Analysis

**DOI:** 10.3390/nu18132098

**Published:** 2026-06-26

**Authors:** Dimitrios Rallis, Maria Lithoxopoulou, Efstratios Saliakellis, Theodora Delaporta, Konstantina Kapetaniou, Eftychia Drogouti, Georgios Kerpiniotis, Evangelia Aggeli, Athina Goulordava, Christos Tsakalidis

**Affiliations:** 1 Second Neonatal Unit and Department of Neonatology, Faculty of Medicine, Aristotle University of Thessaloniki, 56403 Thessaloniki, Greece; mlithoxopoulou@yahoo.com (M.L.); dvradelaporta@gmail.com (T.D.); edrogouti@yahoo.com (E.D.); geokerp@auth.gr (G.K.); elinaangeli.ea@gmail.com (E.A.); athina_8783@hotmail.com (A.G.); tsakalidisx@gmail.com (C.T.); 2 Fourth Department of Pediatrics, Faculty of Medicine, Aristotle University of Thessaloniki, 56403 Thessaloniki, Greece; esalia@auth.gr (E.S.); kapetaniouk@gmail.com (K.K.)

**Keywords:** body composition, feeding practices, human milk, prematurity, weight gain

## Abstract

**Background/Objectives:** Our aim was to assess the benefits and risks of fortified vs. unfortified human milk in preterm/low birth weight neonates and to incorporate the INSPECT-SR tool to enhance evidence appraisal. **Methods:** PubMed and Scopus were searched from inception to 30 November 2025, limited to human studies published in English. Randomized controlled trials in neonates ≤ 34 weeks’ gestation or ≤2500 g birth weight were included. Data extraction followed PRISMA guidelines. Risk of bias was assessed using Cochrane RoB 2, and trustworthiness using the INSPECT-SR tool. Random-effects models were used to pool mean differences (MDs) and odds ratios (ORs); heterogeneity was assessed with I^2^. Outcomes included weight, length, and head circumference gain velocities, feeding intolerance, necrotizing enterocolitis, late-onset sepsis, chronic lung disease, time to full enteral feeding, length of stay, and mortality. **Results:** Fifteen randomized controlled trials involving 1079 neonates (546 receiving fortified human milk and 533 receiving unfortified human milk) were included in the analysis. Fortified human milk was associated with significantly greater weight gain (MD 2.74 [1.27–4.22] g/kg/d; I^2^ = 95%), length gain (MD 0.08 [0.02–0.14] cm/week; I^2^ = 90%), and head circumference gain (MD 0.06 [0.03–0.10] cm/week; I^2^ = 87%). No significant differences were observed in feeding intolerance, necrotizing enterocolitis, late-onset sepsis, chronic lung disease, time to full enteral feeding, length of stay, or mortality. Subgroup analyses suggested that the publication era partially contributed to heterogeneity, although substantial residual heterogeneity remained. According to INSPECT-SR, two trials were judged as having no concerns, twelve as having some concerns, and one as having serious concerns regarding trustworthiness. **Conclusions:** Low-certainty evidence suggests that human milk fortification improves short-term anthropometric growth parameters in preterm and low birth weight infants without increasing major neonatal morbidities. However, the certainty of these findings is limited by substantial heterogeneity and concerns regarding study trustworthiness. Future adequately powered trials using contemporary fortification strategies and long-term follow-up are required to establish the clinical significance of these growth benefits.

## 1. Introduction

Long-term health outcomes in preterm infants are closely associated with early nutritional practices [1]. Furthermore, the clinical benefits of human milk for preterm infants are well established [2,3]. However, previous research has shown that preterm infants fed exclusively on human milk often exhibit slower postnatal growth and lower weight-for-age z-scores from birth to hospital discharge compared with those receiving formula-based nutrition [4,5]. As preterm infants have significantly higher caloric and protein needs than term infants to approximate fetal growth rates, inadequate nutrient provision—particularly protein—remains a persistent challenge [6]. Human milk fortification has therefore become a key strategy to promote catch-up growth and support the accrual of weight, length, and head circumference while maintaining appropriate body composition in preterm infants. Before the routine use of human milk fortifiers, growth in human-milk-fed preterm infants lagged behind that of infants fed with preterm formula [7]. Although fortification has improved nutrient delivery, contemporary studies still report mixed findings regarding its impact on growth parameters [8,9]. Consequently, clinicians must balance the prevention of postnatal growth restriction against the potential risks associated with alternative feeding strategies (e.g., preterm formula), including late-onset sepsis, necrotizing enterocolitis, retinopathy of prematurity, and potential longer-term developmental consequences such as cognitive and motor outcomes [5,10].

Although several systematic reviews and meta-analyses have previously examined the effects of human milk fortification in preterm and low birth weight infants [11,12], these studies have largely focused on pooled efficacy outcomes without systematically evaluating the internal validity and trustworthiness of the included trials beyond conventional risk-of-bias frameworks. The Investigating Problematic Clinical Trials in Systematic Reviews (INSPECT-SR) tool [13] is a recently developed methodological instrument designed to evaluate the trustworthiness of randomized controlled trials included in systematic reviews. Unlike conventional risk-of-bias tools, which primarily assess internal validity, INSPECT-SR provides a structured framework to identify concerns related to research conduct, governance, transparency, and data integrity. It systematically examines domains such as trial registration, ethical approval, plausibility of recruitment and methodology, consistency of reported results, and the presence of post-publication notices [13]. By capturing potential issues that may not be detected through standard appraisal methods, INSPECT-SR enhances the critical evaluation of evidence and supports more reliable interpretation of meta-analytic findings.

In this context, the present study aims to examine the evidence regarding the benefits and risks of fortified human milk compared with unfortified human milk in preterm or low birth weight neonates and introduces an additional methodological dimension by incorporating the INSPECT-SR tool [13]. By integrating this approach alongside standard risk-of-bias assessment, this meta-analysis aims not only to quantify clinical effects but also to strengthen the interpretability and credibility of the synthesized evidence.

## 2. Materials and Methods

Two authors (T.D. and M.L.) formulated a pre-specified search protocol, according to the population, intervention comparison, and outcome (PICO) worksheet (Table A1). The protocol was registered in PROSPERO (CRD42025642901). The Preferred Reporting Items for Systematic reviews and Meta-Analyses (PRISMA) guidelines were adopted for this systematic review [14]. Eligibility criteria were as follows: studies published from inception to 30 November 2025; human studies published in English; and studies providing full-text access and sufficient outcome data for extraction. Both randomized and observational studies were initially considered eligible; however, no observational studies met the predefined inclusion criteria. Review articles, case series, case–control studies, and opinion articles not reporting original data were excluded. Non-human studies, studies in other languages, studies that examined the effect of human milk fortification compared with formula, or studies that examined the effect of human milk fortification on neonates > 34 weeks of gestational age or birth weight > 2500 g were excluded. Moreover, studies including neonates with severe genetic or chromosomal abnormalities that could affect growth were also excluded.

### 2.1. Search Strategy

Two authors (T.D. and M.L.) independently performed a literature search in the PubMed and Scopus databases. The literature search was conducted from inception to 30 November 2025, with the following filters: ((premature) OR (preterm) OR (low birth weight) OR (LBW)) AND (neonate) AND ((breastfeeding) OR (human milk fortification) AND (clinical outcomes)). The full search strategy is presented in Table A2. The references of the identified studies were also searched to ensure that no study was missed.

For the purposes of this review, fortification was broadly defined as the addition of nutrients or energy-containing supplements to human milk with the aim of improving nutritional intake and growth. This definition encompasses bovine multinutrient fortifiers as well as human fortifiers. Studies evaluating non-standard fortification strategies were identified and assessed separately but were not included in the quantitative meta-analysis because of their distinct biological mechanisms. One study evaluated coconut oil supplementation rather than conventional multinutrient fortification [15]. Although coconut oil increases caloric density, it does not provide the protein, minerals, and micronutrients supplied by standard human milk fortifiers. Consequently, the study [15] was excluded from the review and meta-analysis.

### 2.2. Screening Process and Study Selection

Both authors (T.D. and M.L.) independently screened the abstracts of all studies identified for eligibility using Rayyan [16] and independently reviewed the full text of each preliminarily included study to confirm eligibility. Any conflicts were resolved by a third reviewer (D.R.).

### 2.3. Extraction of Data from Selected Studies: Components of the Data Extraction Form

Data were extracted by two authors (T.D. and M.L.) independently on a Microsoft Excel spreadsheet that was piloted prior to use. Extracted data included, but were not limited to, author name, year, journal name, country where the study was conducted, study methodology and design, case population, comparative group, sample size, and primary and secondary outcomes.

### 2.4. Quality Assessment

The Cochrane risk of bias (RoB 2) for clinical trials was used for the evaluation of the methodological quality of included clinical trials [17]. Two independent researchers (T.D. and M.L.) assessed each study using the five standard RoB 2 domains: (1) bias arising from the randomization process, (2) bias due to deviations from intended interventions, (3) bias due to missing outcome data, (4) bias in measurement of the outcome, and (5) bias in selection of the reported result. For each domain, signaling questions were answered according to the RoB 2 algorithm, leading to judgments of “low risk of bias,” “some concerns,” or “high risk of bias.” An overall risk-of-bias judgment for each outcome was then derived following the Cochrane handbook guidance. Any discrepancies between reviewers were resolved through discussion and, when necessary, consultation with a third reviewer (D.R.).

### 2.5. Sensitivity Analysis

A trustworthiness assessment according to the INSPECT-SR tool [13] was performed in all included studies, independently by two researchers (T.D. and M.L.); a third investigator (D.R.) resolved any existing discrepancies.

Moreover, a subgroup analysis was performed for the outcomes with heterogeneity > 75% based on publication era (between 2000 and 2025 and between 1986 and 1999). Also, we examined the small study effect by performing a subgroup analysis of the studies that included more than 20 individuals.

### 2.6. Analysis and Data Synthesis

A quantitative synthesis was performed to measure the effect of human milk fortification on neonatal growth, along with the precision of the effect estimate. For studies reporting only per-protocol outcome data, effect estimates were extracted as reported because insufficient information was available to reconstruct intention-to-treat analyses. Effect estimates from individual studies were weighted and combined, with forest plots generated for any outcome reported by a minimum of two studies (Review Manager Version 5.4, The Cochrane Collaboration, 2020). Mean differences (MDs) and odds ratios (ORs) were calculated for neonatal subgroups using a random-effects model [18]. Heterogeneity was assessed with the I^2^ statistic [19], statistical significance was set at *p* < 0.05, and publication bias was tested with Egger’s method in JASP software (version 0.97.0) [20].

### 2.7. Certainty of Evidence

The certainty of evidence was assessed independently by two researchers (T.D. and M.L.) according to the Grading of Recommendations Assessment, Development and Evaluation (GRADE) framework [21], across the domain of risk of bias, inconsistency, indirectness, imprecision, and publication bias.

## 3. Results

**Figure 1:**
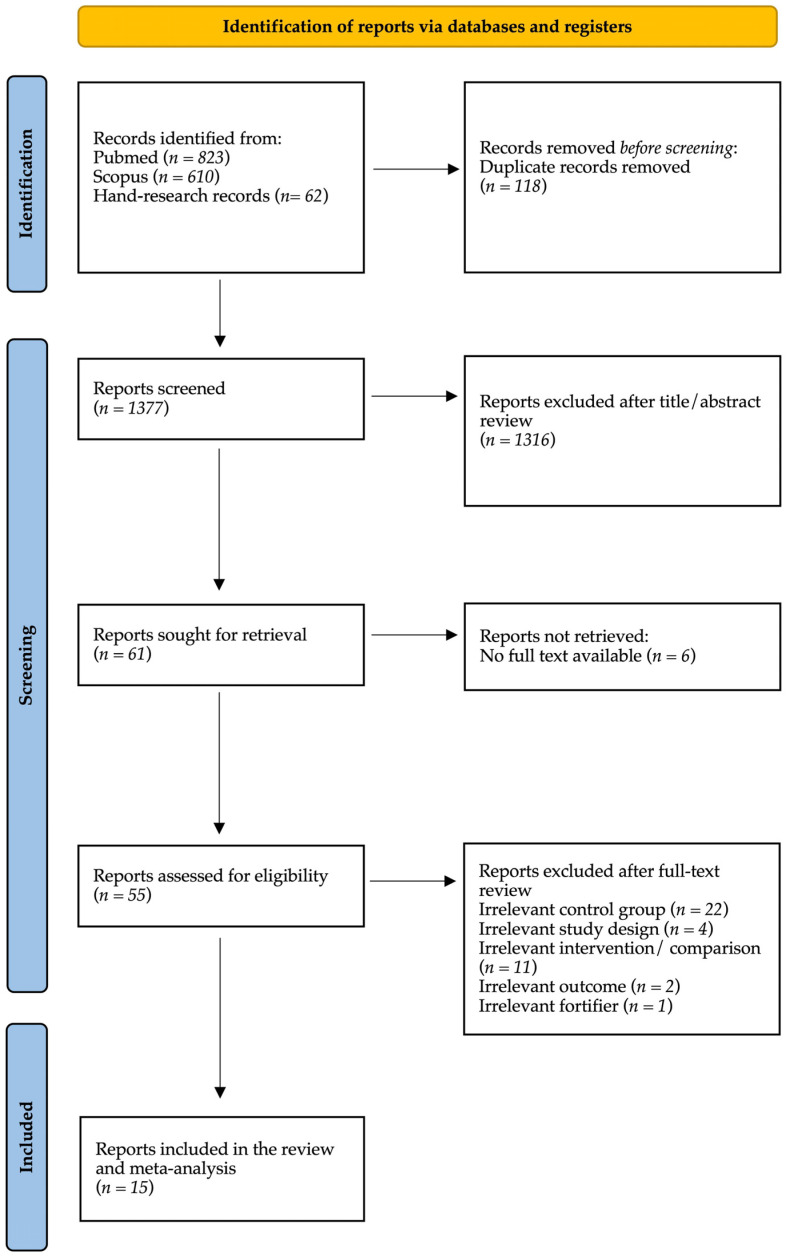
PRISMA flow chart for study selection.

The literature search identified 1495 reports, of which 61 reports were sought for retrieval; finally, 55 studies were assessed for eligibility. After a full-text review, 15 studies [22,23,24,25,26,27,28,29,30,31,32,33,34,35,36] were included in the systematic review and meta-analysis (Figure 1). All studies were randomized controlled trials published between 1986 and 2023; no eligible studies published in 2024 or 2025 met the inclusion criteria despite the search being conducted through November 2025. Although observational studies were considered eligible during protocol development, no eligible non-randomized studies were identified. A total of 1079 neonates were included; 546 received fortified human milk, whereas 533 received unfortified human milk. Seven studies [22,23,24,27,28,30,32] were published between 2000 and 2025, whereas eight studies were published between 1986 and 1999 [25,26,29,31,33,34,35,36]. Finally, 11 studies [22,23,24,27,28,29,30,32,33,34,36] included more than 20 neonates. Individual studies’ characteristics and main outcomes are depicted in Table A3 and Table A4.

**Figure 2:**
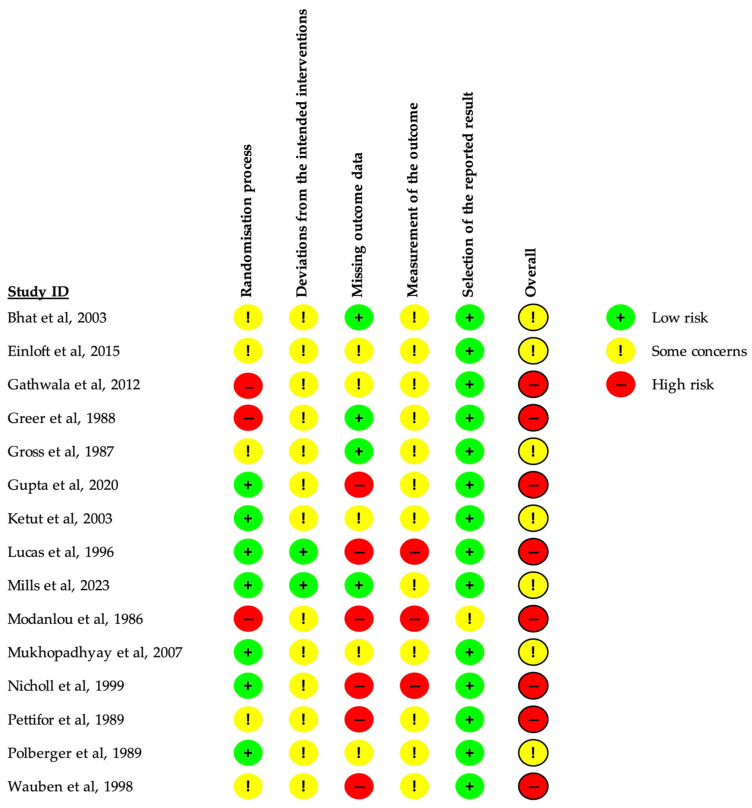
Risk of bias (ROB)-2 assessment of the included studies.

**Figure A1:**
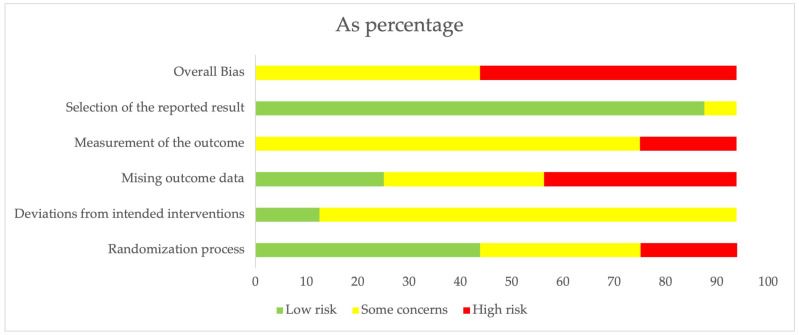
Summary of the risk of bias (ROB)-2 assessment of the included studies.

Quality assessment revealed that eight studies [24,25,27,29,31,33,34,36] were at high risk of bias, mainly due to randomization, missing outcome data, or outcome measurement concerns, whereas seven studies were judged to have some concerns [22,23,26,28,30,32,35] (Figure 2 and Figure A1).

### 3.1. Sensitivity Analysis

**Figure A2:**
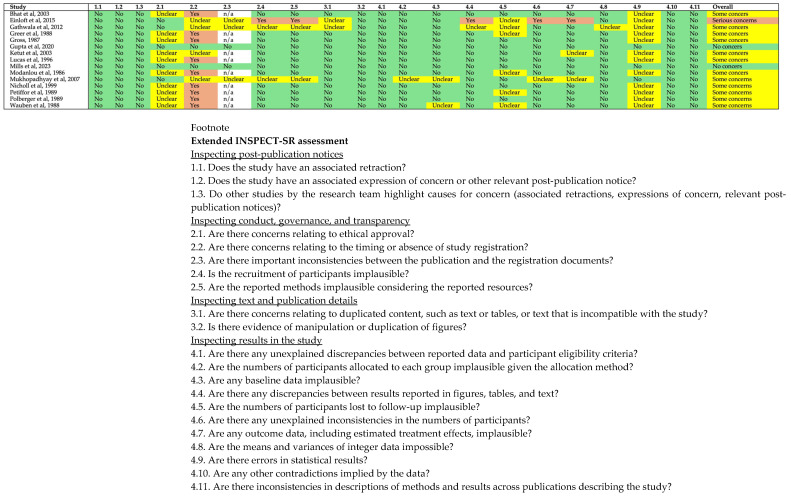
Extended INSPECT-SR assessment of the included studies.

Based on the trustworthiness assessment of the included studies, no post-publication notices were identified. Ten trials [22,25,26,28,29,31,33,34,35,36] lacked an explicitly reported protocol or trial registration number and provided limited detail on sequence generation and allocation concealment; these transparency and reporting gaps led to some concerns in the conduct/governance domain. INSPECT-SR identified substantial concerns regarding trial conduct and reporting transparency in a study [23], due to post-randomization exclusions and the absence of allocation concealment because selective (intentionally or not) inclusion of participants was allowed. In inspecting results and publication details, the judgment raised some concerns in two studies [23,24]; in the remaining studies, text, tables, and numerical results were internally consistent, and outcome magnitudes were biologically plausible. Statistical analysis and incomplete pre-specification/reporting created some concerns about conduct/transparency and result robustness in 12 studies [22,23,24,25,26,28,29,31,33,34,35,36]; however, there were no clear data inconsistencies or signs of manipulation. INSPECT-SR also identified substantial concerns in a study [23], due to retrospective post-randomization exclusions based on clinical status, respiratory support, parenteral nutrition duration, feeding adequacy, and availability of scans, which resulted in only 38 infants (out of 83 randomized infants) being included in the final analysis. These post-randomization exclusions, lack of transparency regarding allocation procedures, and discrepancies in reported results led to the judgment of important concerns regarding trustworthiness. Overall, following INSPECT-SR guidance, 2 trials received a judgement of “no concerns” [27,30], 12 trials received an overall judgement of “some concerns” [22,24,25,26,28,29,31,32,33,34,35,36], and 1 trial received an overall judgement of “serious concerns” [23] (Figure A2).

### 3.2. Publication Bias

From the Egger’s test, there was a low suspicion of publication bias for all outcomes examined, i.e., weight gain, length gain, feeding intolerance, necrotizing enterocolitis, late-onset sepsis, length of stay, and time to full enteral feeds, except for the head circumference gain where a publication bias was detected (Table A5).

### 3.3. Effects of Fortified Compared with Unfortified Human Milk on Weight, Length, and Head Circumference Gain

**Figure 3:**
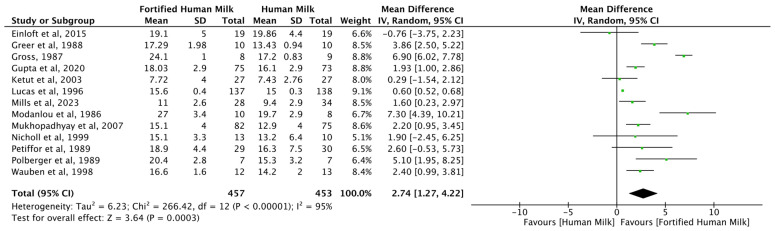
Forest plot of the meta-analysis on weight gain.

Thirteen studies examined the effects of fortified compared with unfortified human milk on weight gain [23,25,26,27,28,29,30,31,32,33,34,35,36]. The meta-analysis of those 13 studies revealed a significantly (Z = 3.64, *p* = 0.0003) higher weight gain velocity (MD 2.74 [1.27–4.22] g/kg/d) in neonates who received fortified compared with those who received unfortified human milk, with heterogeneity I^2^ = 95% (Figure 3). After removing the study by Einloft et al. [23], a significantly (Z = 3.80, *p* = 0.0001) higher weight gain velocity (MD 2.99 [1.45–4.54] g/kg/d) was noted in neonates who received fortified compared with those who received unfortified human milk, with heterogeneity I^2^ = 96%.

**Figure 4:**
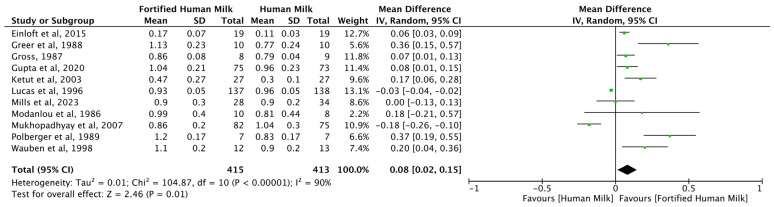
Forest plot of the meta-analysis on length gain.

Based on the meta-analysis of 11 studies [23,25,26,27,28,29,30,31,32,35,36], length gain velocity was significantly (Z = 2.46, *p* = 0.01) higher among neonates who received fortified compared with those who received unfortified human milk (MD 0.08 [0.02–0.15] cm/week), with heterogeneity I^2^ = 90% (Figure 4). After removing the study by Einloft et al. [23], a significant difference (Z = 2.24, *p* = 0.03) in length gain velocity (MD 0.09 [0.01–0.17] cm/week) was noted between the two groups, with heterogeneity I^2^ = 89%.

**Figure 5:**
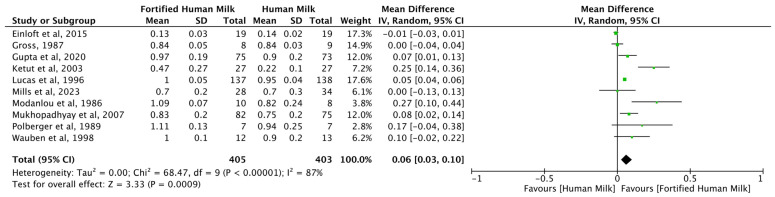
Forest plot of the meta-analysis on head circumference gain.

Finally, the meta-analysis of 10 studies [23,26,27,28,29,30,31,32,35,36] revealed a significantly (Z = 3.33, *p* = 0.0009) higher head circumference gain velocity (MD 0.06 [0.03–0.10] cm/week) in neonates who received fortified compared with those who received unfortified human milk, with heterogeneity I^2^ = 87% (Figure 5). After removing the study by Einloft et al. [23], a significantly (Z = 3.75, *p* = 0.0002) higher head circumference gain velocity (MD 0.08 [0.04–0.12] cm/week) was noted in neonates who received fortified compared with those who received unfortified human milk, with heterogeneity I^2^ = 73%.

### 3.4. Effects of Fortified Compared with Unfortified Human Milk on Secondary Outcomes

**Figure A3:**
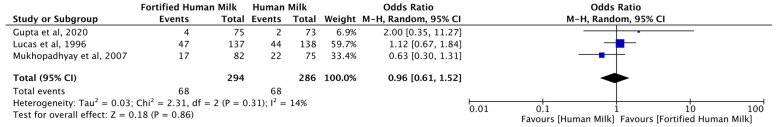
Forest plot of the meta-analysis on feeding intolerance.

**Figure A4:**
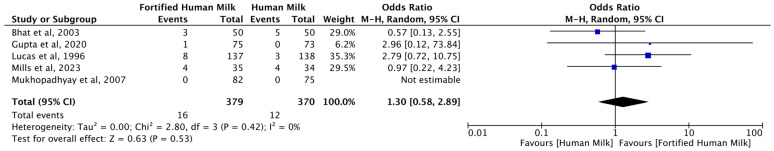
Forest plot of the meta-analysis on necrotizing enterocolitis.

**Figure A5:**
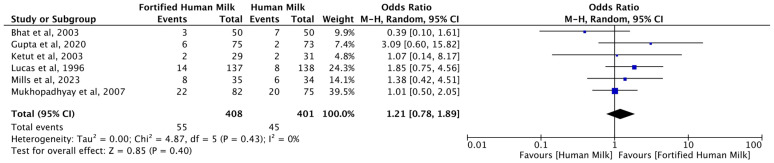
Forest plot of the meta-analysis on late-onset sepsis.

**Figure A6:**
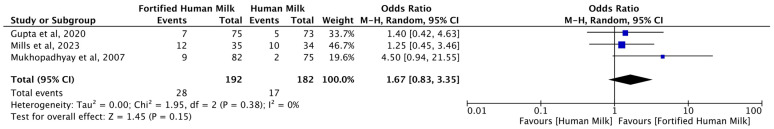
Forest plot of the meta-analysis on chronic lung disease.

Based on the meta-analysis of three studies [27,29,32], five [22,27,29,30,32], and six studies [22,27,28,29,30,32], no significant difference was observed in feeding intolerance (Z = 0.18, *p* = 0.86), necrotizing enterocolitis (Z = 0.63, *p* = 0.53), or late-onset sepsis (Z = 0.85, *p* = 0.40) between neonates who received fortified and those who received unfortified human milk (Figure A3, Figure A4 and Figure A5). Also, the meta-analysis of three studies [27,30,32] revealed no significant difference in the incidence of chronic lung disease (Z = 1.45, *p* = 0.15) between neonates who received fortified and those who received unfortified human milk, with heterogeneity I^2^ = 0% (Figure A6).

**Figure A7:**
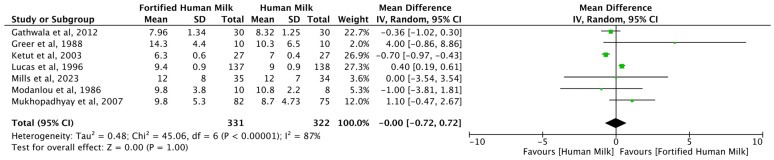
Forest plot of the meta-analysis on time to full enteral feeding.

**Figure A8:**
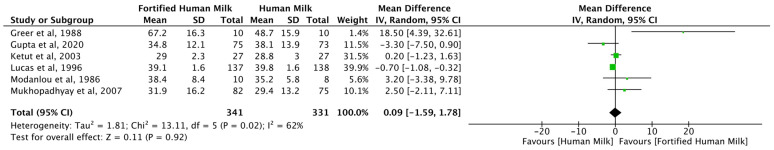
Forest plot of the meta-analysis on length of stay.

**Figure A9:**
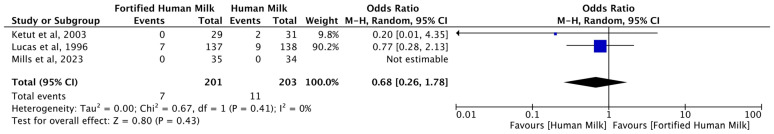
Forest plot of the meta-analysis on mortality.

Regarding the remaining secondary outcomes, no significant differences were revealed in the mean difference of the time to full enteral feeding, and length of stay between neonates who received fortified and those who received unfortified human milk, based on the meta-analysis of seven [24,25,28,29,30,31,32] (Figure A7) and six studies [25,27,28,29,31,32] (Figure A8), respectively. Finally, no significant difference was revealed in mortality between neonates who received fortified and those who received unfortified human milk, based on the meta-analysis of three studies [28,29,30] (Figure A9).

### 3.5. Certainty of Evidence

According to GRADE, the certainty of evidence for anthropometric and secondary outcomes ranged from very low to low, primarily because of substantial inconsistency (I^2^ > 75%) and methodological limitations of several included trials (Table A6 and Table A7).

### 3.6. Subgroup Analysis Based on Publication Era

**Figure A10:**
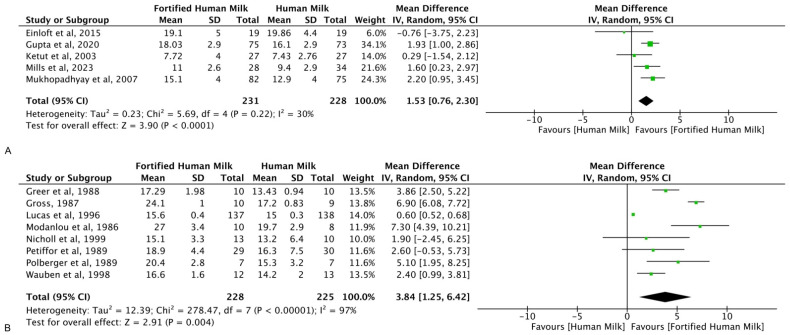
(**A**) Forest plot of the meta-analysis on weight gain among studies that provided extractable data and were published between 2000 and 2023. (**B**) Forest plot of the meta-analysis on weight gain among studies that provided extractable weight gain data and were published between 1986 and 1999.

In the subgroup analysis, the meta-analysis of the five studies [23,27,28,30,32] between 2000 and 2025 revealed a significantly (Z = 3.90, *p* < 0.0001) higher weight gain velocity (MD 1.53 [0.76–2.30] g/kg/d) in neonates who received fortified compared with those who received unfortified human milk, with heterogeneity I^2^ = 30% (Figure A10A). Two studies [22,24] published after 2000 did not provide extractable weight-gain velocity data and therefore could not contribute to the corresponding subgroup analysis. After removing the study by Einloft et al. [23], a significantly (Z = 5.36, *p* < 0.00001) higher weight gain velocity (MD 1.74 [1.10–2.37] g/kg/d) was noted in neonates who received fortified compared with those who received unfortified human milk, with heterogeneity I^2^ = 4%. Moreover, the meta-analysis of the eight studies [25,26,29,31,33,34,35,36] between 1986 and 1999 revealed a significantly (Z = 2.91, *p* < 0.004) higher weight gain velocity (MD 3.84 [1.25–6.42] g/kg/d) in neonates who received fortified compared with those who received unfortified human milk, with heterogeneity I^2^ = 97% (Figure A10B).

**Figure A11:**
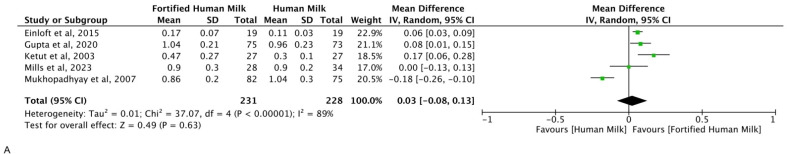

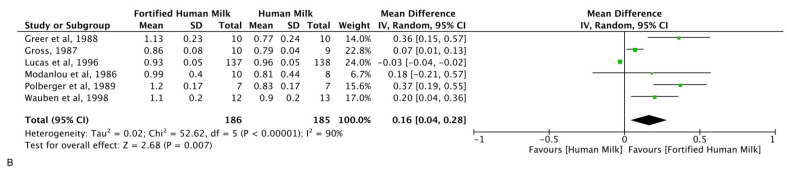
(**A**) Forest plot of the meta-analysis on length gain among studies that provided extractable data and were published between 2000 and 2023. (**B**) Forest plot of the meta-analysis on length gain among studies that provided extractable data and were published between 1986 and 1999.

The meta-analysis of the five studies [23,27,28,30,32] between 2000 and 2025 revealed no significant difference (Z = 0.49, *p* = 0.63) in length gain velocity among neonates who received fortified compared with those who received unfortified human milk (Figure A11A). The results of the meta-analysis were not affected after removing the study by Einloft et al. [21]. The meta-analysis, however, of the six studies [25,26,29,31,35,36] between 1986 and 1999 revealed a significantly (Z = 2.68, *p* = 0.007) higher length gain velocity (MD 0.16 [0.04–0.28] cm/week) in neonates who received fortified compared with those who received unfortified human milk, with heterogeneity I^2^ = 90% (Figure A11B).

**Figure A12:**
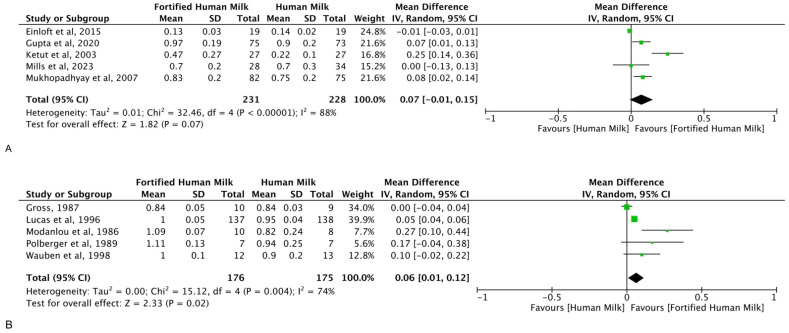
(**A**) Forest plot of the meta-analysis on head circumference gain among studies that provided extractable data and were published between 2000 and 2023. (**B**) Forest plot of the meta-analysis on head circumference gain among studies that provided extractable data and were published between 1986 and 1999.

Also, the meta-analysis of the five studies [23,27,28,30,32] between 2000 and 2025 revealed no significant difference (Z = 1.82, *p* = 0.07) in head circumference gain velocity among neonates who received fortified compared with those who received unfortified human milk (Figure A12A). After removing the study by Einloft et al. [23], a significantly (Z = 2.42, *p* = 0.02) higher head circumference gain velocity (MD 0.10 [0.02–0.18] cm/week) was noted in neonates who received fortified compared with those who received unfortified human milk, with heterogeneity I^2^ = 72%. The meta-analysis of the five studies [26,29,31,35,36] between 1986 and 1999 revealed a significantly (Z = 2.33, *p* = 0.02) higher head circumference gain velocity (MD 0.06 [0.01–0.12] cm/week) in neonates who received fortified compared with those who received unfortified human milk, with heterogeneity I^2^ = 74% (Figure A12B).

Finally, a subgroup analysis was performed regarding the feeding intolerance outcome; the meta-analysis of the studies between 2000 and 2025 and between 1986 and 1999 revealed no significant difference in the odds ratios of feeding intolerance among neonates who received fortified compared with those who received unfortified human milk.

### 3.7. Subgroup Analysis Based on the Sample Size of the Studies

**Figure A13:**
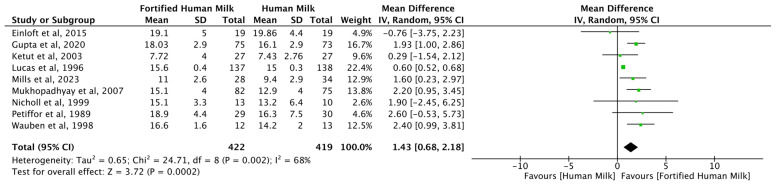
Forest plot of the meta-analysis on weight gain among studies including more than 20 individuals.

The meta-analysis of the nine studies [23,27,28,29,30,32,33,34,36] with a sample size of more than 20 individuals revealed a significantly (Z = 3.72, *p* = 0.0002) higher weight gain velocity (MD 1.43 [0.68–2.18] g/kg/d) in neonates who received fortified compared with those who received unfortified human milk, with heterogeneity I^2^ = 68% (Figure A13)

**Figure A14:**
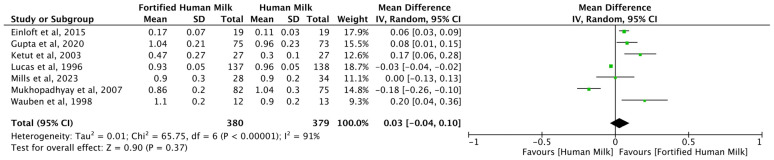
Forest plot of the meta-analysis on length gain among studies including more than 20 individuals.

The meta-analysis of the seven studies [23,27,28,29,30,32,36] with a sample size of more than 20 individuals revealed no significant difference (Z = 0.90, *p* = 0.37) in length gain velocity in neonates who received fortified compared with those who received unfortified human milk (Figure A14).

**Figure A15:**
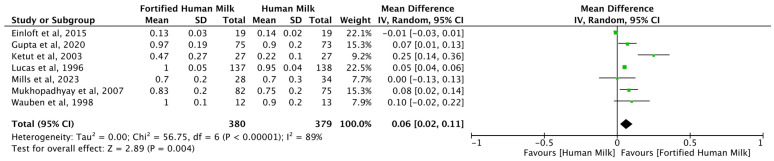
Forest plot of the meta-analysis on head circumference gain among studies including more than 20 individuals.

Finally, the meta-analysis of the seven studies [23,27,28,29,30,32,36] with a sample size of more than 20 individuals revealed a significantly (Z = 2.89, *p* = 0.004) higher head circumference gain velocity (MD 0.06 [0.02–0.11] cm/week) among neonates who received fortified compared with those who received unfortified human milk, with heterogeneity I^2^ = 89% (Figure A15). The results of the meta-analysis were not affected in any of the outcomes after removing the study by Einloft et al. [23].

## 4. Discussion

The findings of this systematic review and meta-analysis revealed that, according to low-certainty evidence, providing fortified rather than unfortified human milk to preterm low birth weight infants leads to an increase in weight, length, and head circumference gain during the early neonatal period. No significant differences were observed in feeding intolerance, late-onset sepsis, necrotizing enterocolitis, chronic lung disease, the time needed to achieve full enteral feeds, length of stay, or mortality between neonates who received fortified compared with unfortified human milk. The GRADE assessment indicated that the certainty of evidence for anthropometric outcomes was low primarily because of substantial inconsistency and methodological limitations of the included trials. The certainty of evidence for major neonatal morbidities was additionally limited by imprecision arising from the low number of events and small sample sizes.

Our findings, in line with a previous Cochrane meta-analysis [12], suggest that multinutrient fortification confers modest improvements in early in-hospital weight, length, and head circumference gain. Of note, in both our and previous meta-analyses, the effect sizes were small and the heterogeneity was high. Even when we removed the trial raising serious concerns according to INSPECT-SR from the analysis, the overall direction of effect remained unchanged in most of the outcomes. The high heterogeneity observed in our meta-analysis is likely attributable to the wide temporal span of the included studies and the differences in the fortifiers utilized. Subgroup analyses based on publication era partially reduced heterogeneity for some outcomes; however, substantial residual heterogeneity remained, suggesting that additional factors such as differences in nutrient composition, feeding protocols, baseline nutritional status, and outcome assessment methods likely contributed to between-study variability. Although subgroup analyses partially explained heterogeneity, the limited number of studies precluded formal meta-regression analyses. More recent studies demonstrated substantially lower heterogeneity for weight gain outcomes compared with older studies, likely reflecting improvements in fortifier composition, nutritional practices, and trial methodology. Early fortifiers used in the 1980s had different compositions and nutrient densities compared with modern fortifiers, which could explain variability in growth outcomes across trials. The clinical relevance of this modest increase should be considered when weighing the benefits against the costs and potential risks of universal fortification. Although statistically significant, the magnitude of the observed improvements was relatively small, and whether these differences translate into clinically meaningful long-term developmental advantages remains uncertain.

Moreover, all studies included in this review evaluated bovine-derived fortifiers, whereas evidence regarding human-milk-derived fortifiers remains comparatively limited. Human-milk-derived fortifiers have attracted increasing interest because they reduce exposure to bovine-derived proteins and may theoretically improve gastrointestinal tolerance and preserve the protective biological properties of human milk. However, available randomized evidence remains insufficient to establish clear superiority over bovine-derived products with respect to growth, feeding tolerance, necrotizing enterocolitis, or long-term developmental outcomes. Additional adequately powered comparative trials are required to determine whether human-milk-derived fortifiers provide clinically meaningful advantages.

Optimizing nutritional strategies for very preterm infants remains challenging. Recent trials have compared donor milk, fortified human milk, and preterm formula as supplements to the mother’s own milk. These studies consistently reported faster short-term growth with formula compared with donor milk, and with preterm formula compared with unfortified human milk, but failed to demonstrate differences in long-term anthropometry or neurodevelopment [30,37]. The PREMFOOD trial comparing unfortified human milk, fortified human milk, and preterm formula supplementation until 35 weeks post-menstrual age, suggested that infants receiving preterm formula demonstrated greater weight gain by 35 weeks post-menstrual age; however, MRI-derived body composition and anthropometry at term did not differ across groups [30]. Observational evidence also suggests that predominant or exclusive fortified human milk feeding may be associated with lower fat mass and higher fat-free mass at term compared with predominantly formula-fed infants [38]. Studies investigating early fortification strategies have likewise shown limited differences in body composition at term [39]. Salas et al. showed that extremely preterm infants randomized to human-milk-derived early fortified human milk versus early unfortified human milk (followed by bovine-derived fortified human milk once full feeds were achieved) had similar fat-free mass at term, although the early fortified human milk group exhibited more favorable gains in length and head circumference z-scores [40].

An important contribution of the present study lies in the application of the INSPECT-SR tool [13] as a complementary framework to conventional risk-of-bias assessment. While prior meta-analyses in this field have relied predominantly on methodological quality appraisal [11,12], the incorporation of a structured trustworthiness evaluation enabled the identification of additional concerns related to reporting transparency, post-randomization exclusions, and internal data consistency. This approach provides a more nuanced interpretation of the evidence base, highlighting that the robustness of pooled estimates may be influenced not only by classical sources of bias but also by potential concerns related to reporting transparency and study conduct. Consequently, the findings of this meta-analysis should be interpreted considering both methodological quality and study trustworthiness, underscoring the need for improved reporting standards and rigor in future randomized trials in neonatal nutrition. It should be noted that most INSPECT-SR ratings classified as “some concerns” were related to reporting transparency and administrative issues commonly encountered in older trials, including the absence of prospective trial registration, limited reporting of allocation concealment procedures, and lack of published protocols. Importantly, these concerns generally did not reflect implausible biological effects, internal data inconsistencies, or evidence of research misconduct. Most studies reported outcome estimates that were biologically plausible and internally consistent. We therefore believe that the INSPECT-SR findings primarily highlight limitations in historical reporting standards rather than fundamental concerns regarding the validity of the observed growth outcomes.

Among the limitations of our study, it should be noted that these findings underscore the persistent uncertainties regarding optimal fortification practices. Standard fixed-dose fortification may inadequately address the wide variability in milk composition, contributing to suboptimal growth in several infants. Future research should include adequately powered trials with long-term follow-up, incorporate early biomarkers predictive of later outcomes (e.g., fecal calprotectin, serum insulin-like growth factor 1), compare fortifier compositions, and evaluate targeted or adjustable strategies tailored to the nutrient profile of individual milk feeds. Also, the reliance on physical growth metrics (weight, length, head circumference, and body composition) without available neurodevelopmental data restricts the ability to draw conclusions about long-term outcomes. Additional work is needed to determine whether human-milk-derived fortifiers offer clinical or cost-effective advantages over bovine-derived products and to clarify the long-term developmental and metabolic consequences of early nutritional interventions. It should also be acknowledged that pooled effect estimates should be interpreted cautiously because of substantial clinical and methodological heterogeneity. Publication bias analyses should be interpreted cautiously because several outcomes included fewer than ten studies, limiting the reliability of Egger’s regression analyses. The GRADE assessment indicated that the certainty of evidence for anthropometric outcomes was reduced; consequently, the observed growth benefits should be interpreted cautiously and should not be assumed to translate into long-term clinical or neurodevelopmental advantages.

Finally, INSPECT-SR is a recently introduced tool, and its performance characteristics and inter-rater reliability in neonatal nutrition research remain incompletely established. In addition, several included trials were conducted during the 1980s and 1990s, before prospective trial registration and contemporary reporting standards became routine. Consequently, some transparency-related domains may disadvantage older studies despite the absence of evidence of misconduct or data manipulation. Future work is required to further evaluate the applicability and reproducibility of INSPECT-SR across different clinical disciplines and historical research periods.

## 5. Conclusions

Low-certainty evidence suggests that human milk fortification may improve short-term weight, length, and head circumference gain in preterm and low birth weight infants without increasing major neonatal morbidities. However, confidence in these estimates is limited by substantial heterogeneity, methodological limitations, and imprecision, and further high-quality randomized controlled trials are required to refine these estimates. Moreover, the identification of additional concerns related to internal validity and trustworthiness underscores the need for improved reporting standards and rigor in future randomized trials in neonatal nutrition.

## Data Availability

No new data were created or analyzed in this study.

## Abbreviations

The following abbreviations are used in this manuscript:

INSPECT-SR	Investigating Problematic Clinical Trials in Systematic Reviews
PRISMA	Preferred Reporting Items for Systematic reviews and Meta-Analyses
RoB	Risk-of-bias
MD	Mean difference
OR	Odds ratio
GRADE	Grading of Recommendations Assessment, Development and Evaluation

## Appendix A

**Table A1:** The PICO worksheet of the systematic review and meta-analysis. PICO, population, intervention, comparison, outcome.

PICO	
Population	Preterm neonates ≤ 34 weeks of gestational age, or low birth weight neonates ≤ 2500 g.
Intervention	Fortification of human milk.
Comparison	Between neonates who received fortified human milk and those received unfortified human milk.
Outcome	The evaluation of the impact of human milk fortification in preterm low birth weight neonates on the growth velocity (weight gain in specific times, e.g., weekly, on discharge, etc.) compared with neonates who received unfortified human milk. Additional outcomes included the development of necrotizing enterocolitis, feeding intolerance, late-onset sepsis, retinopathy of prematurity, and bronchopulmonary dysplasia in neonates receiving fortified human milk compared with those receiving unfortified human milk. Also, secondary outcomes were the length of hospital stay and the long-term neurodevelopmental outcome.

**Table A2:** Literature search strategy.

(“premature birth”[MeSH Terms] OR (“premature”[All Fields] AND “birth”[All Fields]) OR “premature birth”[All Fields] OR “premature”[All Fields] OR “prematurely”[All Fields] OR “prematures”[All Fields] OR “prematurities”[All Fields] OR “prematurity”[All Fields] OR (“preterm”[All Fields] OR “preterms”[All Fields]) OR (“infant, low birth weight”[MeSH Terms] OR (“infant”[All Fields] AND “low”[All Fields] AND “birth”[All Fields] AND “weight”[All Fields]) OR “low birth weight infant”[All Fields] OR (“low”[All Fields] AND “birth”[All Fields] AND “weight”[All Fields]) OR “low birth weight”[All Fields]) OR “LBW”[All Fields]) AND (“infant, newborn”[MeSH Terms] OR (“infant”[All Fields] AND “newborn”[All Fields]) OR “newborn infant”[All Fields] OR “neonatal”[All Fields] OR “neonate”[All Fields] OR “neonates”[All Fields] OR “neonatality”[All Fields] OR “neonatals”[All Fields] OR “neonate s”[All Fields]) AND ((“breast feeding”[MeSH Terms] OR (“breast”[All Fields] AND “feeding”[All Fields]) OR “breast feeding”[All Fields] OR “breastfeeding”[All Fields] OR “breastfeedings”[All Fields] OR “breastfeeders”[All Fields] OR ((“milk, human”[MeSH Terms] OR (“milk”[All Fields] AND “human”[All Fields]) OR “human milk”[All Fields] OR (“human”[All Fields] AND “milk”[All Fields])) AND (“fortification”[All Fields] OR “fortifications”[All Fields]))) AND ((“ambulatory care facilities”[MeSH Terms] OR (“ambulatory”[All Fields] AND “care”[All Fields] AND “facilities”[All Fields]) OR “ambulatory care facilities”[All Fields] OR “clinic”[All Fields] OR “clinic s”[All Fields] OR “clinical”[All Fields] OR “clinically”[All Fields] OR “clinicals”[All Fields] OR “clinics”[All Fields]) AND (“outcome”[All Fields] OR “outcomes”[All Fields]))) Translations premature: “premature birth”[MeSH Terms] OR (“premature”[All Fields] AND “birth”[All Fields]) OR “premature birth”[All Fields] OR “premature”[All Fields] OR “prematurely”[All Fields] OR “prematures”[All Fields] OR “prematurities”[All Fields] OR “prematurity”[All Fields] preterm: “preterm”[All Fields] OR “preterms”[All Fields] low birth weight: “infant, low birth weight”[MeSH Terms] OR (“infant”[All Fields] AND “low”[All Fields] AND “birth”[All Fields] AND “weight”[All Fields]) OR “low birth weight infant”[All Fields] OR (“low”[All Fields] AND “birth”[All Fields] AND “weight”[All Fields]) OR “low birth weight”[All Fields] neonate: “infant, newborn”[MeSH Terms] OR (“infant”[All Fields] AND “newborn”[All Fields]) OR “newborn infant”[All Fields] OR “neonatal”[All Fields] OR “neonate”[All Fields] OR “neonates”[All Fields] OR “neonatality”[All Fields] OR “neonatals”[All Fields] OR “neonate’s”[All Fields] breastfeeding: “breast feeding”[MeSH Terms] OR (“breast”[All Fields] AND “feeding”[All Fields]) OR “breast feeding”[All Fields] OR “breastfeeding”[All Fields] OR “breastfeedings”[All Fields] OR “breastfeeders”[All Fields] OR “breastfeeding’s”[All Fields] human milk: “milk, human”[MeSH Terms] OR (“milk”[All Fields] AND “human”[All Fields]) OR “human milk”[All Fields] OR (“human”[All Fields] AND “milk”[All Fields]) fortification: “fortification”[All Fields] OR “fortifications”[All Fields] clinical: “ambulatory care facilities”[MeSH Terms] OR (“ambulatory”[All Fields] AND “care”[All Fields] AND “facilities”[All Fields]) OR “ambulatory care facilities”[All Fields] OR “clinic”[All Fields] OR “clinic’s”[All Fields] OR “clinical”[All Fields] OR “clinically”[All Fields] OR “clinicals”[All Fields] OR “clinics”[All Fields] outcomes: “outcome”[All Fields] OR “outcomes”[All Fields]

**Table A3:** Characteristics of the studies included in the systematic review and meta-analysis.

Study	Country	Design	Inclusion Criteria	Intervention Group (Fortified HM)	Fortifier	Control Group (Unfortified HM)	End of Study	Outcome
Bhat et al., 2003 [22]	Oman	RCT	VLBW	50	Bovine (Multinutrient fortifier)	50	Until discharge	The morbidities included length of hospital stay, between the two groups and examine the growth rate in a sizable sample of preterm VLBW infants fed only breast milk with or without fortifiers.
Einloft et al., 2015 [23]	Brazil	RCT	VLBW < 1500 g	19	Bovine (Nestle 85)	19	Before discharge (at 2000 g body weight)	The effects of supplemented versus unsupplemented HM on bone mineralization as determined by whole-body DXA and calcium/phosphorus metabolism in a sample of VLBW preterm neonates.
Gathwala et al., 2012 [24]	India	RCT	<1800 g	30	Bovine (Lactodex)	30	Until 2200 g body weight	To evaluate the nutritional benefit of fortified human milk in preterm infants.
Greer et al., 1988 [25]	USA	RCT	<1600 g birth weight, <32 weeks	10	Bovine	10	Until discharge	To investigate whether, in contrast to infants given unfortified preterm HM, adding calcium, phosphorus, and protein to the milk of prematurely breastfeeding women would enhance the rate of bone mineralization and growth. Additionally, that babies given supplemented HM would perform better than those given a specific formula for preterm neonates.
Gross, 1987 [26]	USA	RCT	BW < 1600 g	8	Bovine	9	44 weeks of cGA	To ascertain whether adding minerals to human milk improves preterm infants’ bone mineralization. Additionally, the impact of human milk feedings throughout the newborn period, both with and without mineral supplementation, on the following mineral status (after hospital discharge) was assessed.
Gupta et al., 2020 [27]	India	RCT	<34 weeks and <1500 g	75	Bovine (Simyl LBW)	73	Until 1800 g body weight	The effects of fortifying human milk with infant formula powder versus unfortified human milk on the growth and metabolic markers of preterm VLBW newborns.
Ketut et al., 2003 [28]	Indonesia	RCT	LBW	27	Bovine (SMA/S26 Human Milk Fortifier)	27	In 30 days or until discharge	To compare the weight gain, length, and head circumference increase at the conclusion of the study between LBW newborns fed fortified HM and those fed HM alone. Assessing the growth of the 1000–1499 g and 1500–1999 g subgroup, total calorie and volume intake, days of full feeding achievement, and adverse reactions of the control and intervention groups were the secondary objectives.
Lucas et al., 1996 [29]	UK	RCT	<1850 g	137	Bovine (Enfamil)	138	Until discharge or 2000 g body weight	The term clinical and biochemical safety data on human milk fortification; the Bayley mental and psychomotor development indexes at 18 months corrected age, the developmental quotient at 9 months corrected age.
Mills et al., 2023 [30]	UK	RCT	<32 weeks	34	Bovine (Cow and Gate Nutriprem)	35	Until 35 weeks cGA	To evaluate the anthropometry and MRI body composition at term and term plus six weeks.
Modanlou et al., 1986 [31]	USA	RCT	VLBW infants (≤1500 g)	10	Bovine	8	Until discharge or 1800 g body weight	To determine whether there are any advantages over human milk alone when preterm human milk is enhanced with a special fortifier made to supply nutrients at levels like a commercial preterm newborn formula.
Mukhopadhyay et al., 2007 [32]	India	RCT	≤34 weeks and ≤1500 g	82	Bovine	75	Until 2000 g or full breast feeds	Growth in weight, length, and head circumference up to 2 kg were the main outcome measures. Biochemical indicators (sodium, calcium, phosphate, and alkaline phosphatase up to 2 kg), adverse events (feed intolerance, necrotizing enterocolitis), length of hospital stay, and morbidities (sepsis, patent ductus arteriosus, chronic lung disease, and intraventricular hemorrhage) were the secondary outcome measures.
Nicholl et al., 1999 [33]	UK	RCT	VLBW infants (<1500 g)	13	Bovine	10	Until discharge	Examining the impact of a commercial fortifier on VLBW infants during a 14-month period.
Petiffor et al., 1989 [34]	S. Africa	RCT	VLBW < 1500 g	29	Bovine (Ross Human Milk fortifier)	30	Until 1800 g body weight	The efficacy of a commercial breast-milk fortifier in the feeding of VLBW infants.
Polberger et al., 1989 [35]	Sweden	RCT	BW < 1500 g	7	Bovine (Protein and fat fortifier)	7	For 4 weeks	To obtain protein metabolism indices similar to those reported in developing breastfed term newborns and to get a growth rate equivalent to the intrauterine rate over the duration of the study.
Wauben et al., 1988 [36]	Canada	RCT	Birth weight < 1800 g	12	Bovine (Experimental multinutrient fortifier)	13	Until discharge (>38 weeks)	To ascertain whether adding a novel multinutrient supplement would enhance growth in the short term more than adding calcium and phosphorus alone.

HM, human milk; RCT, randomized controlled trial; VLBW, very low birth weight; MM, maternal milk; cGA, corrected gestational age; DXA, dual energy X-ray absorptiometry.

**Table A4:** Outcomes of the studies included in the systematic review and meta-analysis.

Study	Weight Gain, g/kg/d	Length, cm/w	Head Circumference, cm/w	Feeding Intolerance	Necrotizing Enterocolitis	Sepsis	Chronic Lung Disease	Length of Stay, d	Time to Full Enteral Feeds, d	Total Body Fat Percentage, %	Triceps Skin Fold, cm	Neurodevelopment	Mortality
Bhat et al., 2003 [22]	-	-	-	-	3/50 vs. 5/50	3/50 vs. 7/50	-	-	-	-	-	-	-
Einloft et al., 2015 [23]	19.1 ± 5.0 vs. 19.8 ± 4.4	0.17 ± 0.07 vs. 0.11 ± 0.03	0.13 ± 0.03 vs. 0.14 ± 0.02	-	-	-	-	-	-	-	-	-	-
Gathwala et al., 2012 [24]	-	-		-	-	-	-	-	7.96 ± 1.34 vs. 8.32 ± 1.25	-	-	-	-
Greer et al., 1988 [25]	17.29 ± 1.98 vs. 13.43 ± 0.94	1.13 ± 0.23 vs. 0.77 ± 0.24	1.05 ± 0.16 vs. 0.80 vs. 0.17	-	-	-	-	67.2 ± 16.3 vs. 48.7 ± 15.9	14.3 ± 4.4 vs. 10.3 ± 6.5	-	0.33 ± 0.18 vs. 0.27 ± 0.16/w	-	-
Gross, 1987 [26]	24.1 ± 1.0 vs. 17.2 ± 0.83	0.86 ± 0.08 vs. 0.79 ± 0.04	0.84 ± 0.05 vs. 0.84 ± 0.03	-	-	-	-	-	-	-	-	-	-
Gupta et al., 2020 [27]	18.03 ± 2.9 vs. 16.1 ± 2.9	1.04 ± 0.21 vs. 0.96 ± 0.23	0.97 ± 0.19 vs. 0.9 ± 0.2	4/75 vs. 2/73	1/75 vs. 0/73	6/75 vs. 2/73	7/75 vs. 5/73	34.8 ± 12.1 vs. 38.1 ± 13.9	-	-	-	-	-
Ketut et al., 2003 [28]	7.72 ± 4.0 vs. 7.43 ± 2.76	0.47 ± 0.27 vs. 0.3 ± 0.1	0.47 ± 0.27 vs. 0.22 ± 0.1	-	-	2/29 vs. 2/31	-	29 ± 2.3 vs. 28.8 ± 3.0	6.3 ± 0.6 vs. 7.0 ± 0.4	-	-	-	0/29 vs. 2/31
Lucas et al., 1996 [29]	15.6 ± 0.4 vs. 15.0 ± 0.3	0.93 ± 0.05 vs. 0.96 ± 0.05	1 ± 0.05 vs. 0.95 ± 0.04	47/137 vs. 44/138	8/137 vs. 3/138	14/137 vs. 8/138	-	39.1 ± 1.6 vs. 39.8 ± 1.6	9.4 ± 0.9 vs. 9.0 ± 0.9	-	-	106.0 ± 2.0 vs. 103.8 ± 2.0 (MDI), 92.3 ± 1.6 vs. 89.9 ± 1.5 (PDI)	7/137 vs. 9/138
Mills et al., 2023 [30]	11.0 ± 2.6 vs. 9.4 ± 2.9	0.9 ± 0.3 vs. 0.9 ± 0.2	0.7 ± 0.2 vs. 0.7 ± 0.3	-	4/35 vs. 4/34	8/35 vs. 6/34	12/35 vs. 10/34	-	12 ± 8 vs. 12 ± 7	-	-	-	0/35 vs. 0/34
Modanlou et al., 1986 [31]	27.0 ± 3.4 vs. 19.7 ± 2.9	0.99 ± 0.4 vs. 0.81 ± 0.44	1.09 ± 0.07 vs. 0.82 ± 0.24	-	-	-	-	38.4 ± 8.4 vs. 35.2 ± 5.8	9.8 ± 3.8 vs. 10.8 ± 2.2	-	-	-	-
Mukhopadhyay et al., 2007 [32]	15.1 ± 4 vs. 12.9 ± 4	0.86 ± 0.2 vs. 1.04 ± 0.3	0.83 ± 0.2 vs. 0.75 ± 0.2	17/82 vs. 22/75	0/82 vs. 0/75	22/82 vs. 20/75	9/82 vs. 2/75	31.9 ± 16.2 vs. 29.4 ± 13.2	9.8 ± 5.3 vs. 8.7 ± 4.73	-	-	-	-
Nicholl et al., 1999 [33]	15.1 ± 3.3 vs. 13.2 ± 6.4	-	-	-	-	-	-	-	-	-	-	-	-
Petiffor et al., 1989 [34]	18.9 ± 4.4 vs. 16.3 ± 7.5	-	-	-	-	-	-	-	-	-	-	-	-
Polberger et al., 1989 [35]	20.4 ± 2.8 vs. 15.3 ± 3.2	1.20 ± 0.17 vs. 0.83 ± 0.17	1.11 ± 0.13 vs. 0.94 ± 0.25	-		-	-	-	-	-	-	-	-
Wauben et al., 1988 [36]	16.6 ± 1.6 vs. 14.2 ± 2.0	1.1 ± 0.2 vs. 0.9 ± 0.2	1.0 ± 0.1 vs. 0.9 ± 0.2	-	-	-	-	-	-	21% vs. 21%	-	-	-

MDI, mental developmental index; PDI, psychomotor developmental index.

**Table A5:** The evaluation of the publication bias with Egger’s method.

Outcome	Intercept	Z	*p*-Value
Weight gain	2.543	0.220	0.826
Length gain	0.079	1.195	0.232
Head circumference gain	0.230	2.817	0.005
Feeding intolerance	−0.255	0.752	0.452
Necrotizing enterocolitis	0.117	0.154	0.885
Sepsis	0.786	−1.413	0.679
Length of stay	−0.934	1.316	0.171
Time to full enteral feeds	−0.290	1.009	0.313

**Table A6:** Summary of findings (GRADE) on fortified human milk versus unfortified human milk in preterm/low birth weight infants.

Outcome	Participants (Studies)	Effect Estimate	Certainty of Evidence (GRADE)	Reasons for Downgrading
Weight gain	910 infants (13 RCTs)	MD 2.74 g/kg/day (95% CI 1.27 to 4.22)	⊕⊕⊝⊝ LOW	Serious risk of bias; very serious inconsistency (I^2^ = 95%)
Length gain	828 infants (11 RCTs)	MD 0.08 cm/week (95% CI 0.02 to 0.15)	⊕⊕⊝⊝ LOW	Serious risk of bias; very serious inconsistency (I^2^ = 90%)
Head circumference gain	808 infants (10 RCTs)	MD 0.06 cm/week (95% CI 0.03 to 0.10)	⊕⊝⊝⊝ VERY LOW	Serious risk of bias; very serious inconsistency (I^2^ = 87%); publication bias detected
Feeding intolerance	580 infants (3 RCTs)	OR 0.96 (95% CI 0.61 to 1.52)	⊕⊕⊝⊝ LOW	Serious risk of bias; serious imprecision
Necrotizing enterocolitis	749 infants (5 RCTs)	OR 1.30 (95% CI 0.58 to 2.89)	⊕⊕⊝⊝ LOW	Serious risk of bias; serious imprecision
Sepsis	809 infants (6 RCTs)	OR 1.21 (95% CI 0.78 to 1.89)	⊕⊕⊝⊝ LOW	Serious risk of bias; serious imprecision
Chronic lung disease	374 (3 RCTs)	OR 1.67 (95% CI 0.83 to 3.35)	⊕⊕⊝⊝ LOW	Serious risk of bias; serious imprecision
Time to full enteral feeds	653 (7 RCTs)	MD −0.00 d (95% CI −0.72 to 0.72)	⊕⊝⊝⊝ VERY LOW	Serious risk of bias; very serious inconsistency (I^2^ = 87%); serious imprecision
Length of stay	672 (6 RCTs)	MD 0.09 d (95% CI −1.59 to 1.78)	⊕⊕⊝⊝ LOW	Serious risk of bias; serious imprecision
Mortality	404 (3 RCTs)	OR 0.68 (95% CI 0.26 to 1.78)	⊕⊕⊝⊝ LOW	Serious risk of bias; serious imprecision

**Table A7:** Summary of findings (SoF) on fortified human milk versus unfortified human milk in preterm/low birth weight infants.

Domain	Assessment
Weight gain	
Risk of bias	Serious (–1)
Inconsistency	Very serious (–2) (I^2^ = 95%)
Indirectness	Not serious
Imprecision	Not serious
Publication bias	Undetected
Final certainty	LOW
Length gain	
Risk of bias	Serious (–1)
Inconsistency	Very serious (–2) (I^2^ = 90%)
Indirectness	Not serious
Imprecision	Not serious
Publication bias	Undetected
Final certainty	LOW
Head circumference gain	
Risk of bias	Serious (–1)
Inconsistency	Very serious (–2) (I^2^ = 87%)
Indirectness	Not serious
Imprecision	Not serious
Publication bias	Detected
Final certainty	VERY LOW
Feeding intolerance	
Risk of bias	Serious (–1)
Inconsistency	Not serious (I^2^ = 14%)
Indirectness	Not serious
Imprecision	Serious (–1)
Publication bias	Undetected
Final certainty	LOW
Necrotizing enterocolitis	
Risk of bias	Serious (–1)
Inconsistency	Not serious (I^2^ = 0%)
Indirectness	Not serious
Imprecision	Serious (–1)
Publication bias	Undetected
Final certainty	LOW
Sepsis	
Risk of bias	Serious (–1)
Inconsistency	Not serious (I^2^ = 0%)
Indirectness	Not serious
Imprecision	Serious (–1)
Publication bias	Undetected
Final certainty	LOW
Chronic lung disease	
Risk of bias	Serious (–1)
Inconsistency	Not serious (I^2^ = 0%)
Indirectness	Not serious
Imprecision	Serious (–1)
Publication bias	Undetected
Final certainty	LOW
Time to full enteral feeds	
Risk of bias	Serious (–1)
Inconsistency	Very serious (–2) (I^2^ = 87%)
Indirectness	Not serious
Imprecision	Serious (–1)
Publication bias	Undetected
Final certainty	VERY LOW
Length of stay	
Risk of bias	Serious (–1)
Inconsistency	Not serious (I^2^ = 62%)
Indirectness	Not serious
Imprecision	Serious (–1)
Publication bias	Undetected
Final certainty	LOW
Mortality	
Risk of bias	Serious (–1)
Inconsistency	Not serious (I^2^ = 0%)
Indirectness	Not serious
Imprecision	Serious (–1)
Publication bias	Undetected
Final certainty	LOW
